# Response to: DNA identification by pedigree likelihood ratio accommodating population substructure and mutations

**DOI:** 10.1186/2041-2223-2-7

**Published:** 2011-03-25

**Authors:** Thore Egeland, A Philip Dawid, Julia Mortera, Petter Mostad, Andreas Tillmar

**Affiliations:** 1 Department of Chemistry, Biotechnology and Food Science, The Norwegian University of Life Sciences, PO Box 5003, NO-1432 Aas, Norway; 2 Centre for Mathematical Sciences, University of Cambridge, Cambridge, UK; 3 Dipartimento di Economia, Università Roma Tre, Rome, Italy; 4 Department of Mathematical Sciences, Chalmers University of Technology and University of Gothenburg, Sweden; 5 Department of Forensic Genetics and Forensic Toxicology, The National Board of Forensic Medicine, Sweden

## Abstract

Mutation models are important in many areas of genetics including forensics. This letter criticizes the model of the paper 'DNA identification by pedigree likelihood ratio accommodating population substructure and mutations' by Ge *et al*. (2010). Furthermore, we argue that the paper in some cases misrepresents previously published papers.

Please see related letter: http://www.investigativegenetics.com/content/2/1/8.

## Correspondence

In a paper in *Investigative Genetics*, Ge, Budowle and Chakraborty [1] consider DNA identification by pedigree likelihood ratio (LR). A mutation model 'to accommodate the possibility of false exclusion' is presented. The model is explained on page 5: 'The transmission probability of two identical allele (sic) is 1- μ. The probability of a mutation event with *x *step (sic) (*x *> 0) is

where *α *is the probability of being a one-step mutation and μ is the mutation rate of the locus. Equal probabilities for gaining or losing repeats are assumed.'

Apparently equation (8) does not define a probability distribution since summing over *x *gives

Below we interpret 'Equal probabilities for gaining or losing repeats are assumed' to mean that a scaling factor 1/2 should be inserted on the right hand side of equation (8) since this leads to a proper probability distribution summing to 1.

There are several problems with this model. Most importantly, it allows for alleles with zero or negative repeat numbers which is not meaningful. Furthermore, this may also be a practical problem. For instance, using the mutation model for marker THO1 having allele value of three repeats leads to an allele with a value less than or equal to zero with probability 1.25 × 10^-6^. While this probability, based on parameter values α = 0.95 and μ = 0.001 suggested in [1], is small, it is certainly not negligible. Unreasonable results will occur if the model is applied to a sufficiently large number of cases. The model is, therefore, inconsistent both from a biological and practical point of view. There are several ways of overcoming these inconsistencies. However, reasonable modifications may well lead to models that have already been published and implemented. One example of a consistent formulation is summarized by equation (1) in [2]. This latter model is *stationary *and so population allele frequencies are not altered by the mutation process. The LR will be changed by including extra, irrelevant (untyped), persons if a *non-stationary *model is used. We refer to [3-6] for further information on mutation models and their implementation.

Ge *et al*. [1], when referring to the mutation model in [2] on page 6 of their paper, state that this model is 'not supportable' and criticize the fact that transmission probability is related to allele frequency. But are not allele frequencies merely the stationary distribution of a mutation process, given that selection can be assumed to have a negligible impact for forensic markers?

Ge *et al*. [1] dismiss several published models including those mentioned above. We are not convinced by the arguments presented by them and there appears to be no data in the paper or the referenced papers that can justify the claims. In the absence of convincing data and studies comparing models, forensic scientists will have to rely on biological understanding and their own judgment when it comes to choosing appropriate mutation models. It may also be reasonable to try different models and several have been documented and implemented. The model suggested in [1] is not an alternative as it violates basic principles.

## Competing interests

The authors declare that they have no competing interests.

## Authors' contributions

TE wrote most of the manuscript. All the authors contributed, read and approved the manuscript.

## References

[B1] Ge J Budowle B Chakraborty R DNA identification by pedigree likelihood ratio accommodating population substructure and mutations Investig Genet 2010 1 8 10.1186/2041-2223-1-8 21092343 PMC2990736

[B2] Dawid AP Mortera J Pascali VL Non-fatherhood or mutation? A probabilistic approach to parental exclusion in paternity testing Forensic Sci Int 2001 124 55 61 10.1016/S0379-0738(01)00564-3 11741761

[B3] Egeland T Mostad PF Statistical genetics and genetical statistics: a forensic perspective Scand J Statistics 2002 29 297 307 10.1111/1467-9469.00284

[B4] Buard J Brenner C Jeffreys AJ Evolutionary fate of an unstable human minisatellite deduced from sperm-mutation spectra of individual alleles Am J Hum Genet 2002 70 1038 1043 10.1086/339608 11859482 PMC379099

[B5] Dawid AP Mortera J Pascali V Boxel D van Probabilistic expert systems for forensic inference from genetic markers Scand J of Statistics 2002 29 577 595 10.1111/1467-9469.00307

[B6] Dawid AP Mortera J Vicard P Object-oriented Bayesian networks for complex forensic DNA profiling problems Forensic Sci Int 2007 169 195 205 10.1016/j.forsciint.2006.08.028 17055679

